# The Owen Value of Stochastic Cooperative Game

**DOI:** 10.1155/2014/853128

**Published:** 2014-04-29

**Authors:** Cheng-Guo E, Quan-Lin Li, Shi-Yong Li

**Affiliations:** ^1^School of Economics and Management, Yanshan University, Qinhuangdao 066004, China; ^2^Liren College, Yanshan University, Qinhuangdao 066004, China

## Abstract

We consider stochastic cooperative game and give it the definition of the Owen value, which is obtained by extending the classical case. Then we provide explicit expression for the Owen value of the stochastic cooperative game and discuss its existence and uniqueness.

## 1. Introduction

In classical cooperative game theory, payoffs to coalitions of agents are known with certainty, but in today's business world payoffs to agents are uncertain. Charnes and Granot [1] considered cooperative games in stochastic characteristic function form. These are games where the payoff *V*(*S*) to coalition *S* is allowed to be a random variable. Research on this subject was continued by Charnes and Granot [2, 3] and Granot [4]. Suijs and Borm [5] research a different and more extensive model. They describe allocation of *X*_*S*_(*a*) to the members of coalition *S* as the sum of two parts. The first part is a monetary transfer between the agents and the second part is an allocation of fractions of *X*_*S*_(*a*). Dshalalow and Ke [6] are concerned with an antagonistic stochastic game between two players A and B which finds applications in economics and warfare. Levy [7] considered the two-player zero-sum stochastic games with finite state under the assumption that one or both players observe the actions of their opponent after some time-dependent delay.

The Owen value [8] as an important solution concept in cooperative game theory has been studied by a number of researchers, which shows a vector whose elements are agents' share derived from several reasonable bases. However, the Owen value for stochastic cooperative games has not been discussed yet. In this paper, we consider the Owen value of stochastic cooperative games.

We end this section with a short overview of the rest of the paper. In Section 2 we introduce preliminaries of stochastic cooperative game. Then, in Section 3 we first introduce the notion of Owen value of classical cooperative game and Owen value of the stochastic cooperative games as payoff in Theorem 5. We conclude in Section 4 also sketch and some main lines for future research.

## 2. Notations and Preliminaries

**Definition (see [7])**

A stochastic cooperative game is described by a tuple Γ = (*N*, {*X*_*S*_}_*S*⊆*N*_, {≿_*i*_}_*i*∈*N*_), where *N* is the set of agents, *X*_*S*_ : *S* → *L* ^1^(*R*) is the payoff function of coalition *S*, where {*X*_*S*_} ∈ *L* ^1^(*R*) with finite expectation, and ≿_*i*_ is the preference relation of agent *i* over the set *L* ^1^(*R*) of stochastic payoffs with finite expectation. The class of all cooperative games with stochastic payoffs with agent set *N* is denoted by SG(*N*). An allocation of a stochastic payoff *X*_*S*_ to the agents in coalition *S* is represented by a pair (*d* ^*S*^, *r* ^*S*^) ∈ *R* ^*S*^ × *R* ^*S*^ such that ∑_*i*∈*S*_ *d*_*i*_ ≤ 0 and ∑_*i*∈*S*_ *r*_*i*_ = 1 and *r*_*i*_ ≥ 0 for all agents *i* ∈ *S*. The set of all allocations for coalition *S* is denoted by *Z*(*S*).

Given such a pair (*d* ^*S*^, *r* ^*S*^) where *d* ^*S*^ = {*d*_*i*_ | *i* ∈ *S*} and *r* ^*S*^ = {*r*_*i*_ | *i* ∈ *S*}, agents *i* ∈ *S* receive the stochastic payoff *d*_*i*_ + *r*_*i*_ *X*_*S*_ and we can also define this payoff as (*d* ^*S*^, *r* ^*S*^)_*i*_; that is, (*d* ^*S*^, *r* ^*S*^)_*i*_ = *d*_*i*_ + *r*_*i*_ *X*_*S*_. The second part, *r*_*i*_ *X*_*S*_, describes the fraction of *X*_*S*_ that is allocated to agent *i*. The first part, *d*_*i*_, describes the deterministic transfer payments between the agents. When *d*_*i*_ ≥ 0, agent *i* receives money, while *d*_*i*_ < 0 means that this agent pays money. The purpose of these transfer payments is that the agents compensate among themselves for transfers of random payoffs. The set of all individual rational allocations is denoted by IR(*S*). Then

(1) $$\begin{array}{c} \text{IR}\left(S\right)=\left\{\left(d^{S},r^{S}\right)\in Z\left(S\right)\;|\;\forall_{i\in S}:d_{i}+r_{i}X_{S}{≿}_{i}X_{\left\{i\right\}}\right\}. \end{array}$$

**Definition**

(*d* ^*N*^, *r* ^*N*^) ∈ *Z*(*N*) is called the stochastic payoff vectors of the game Γ if it satisfies ∑_*i*∈*N*_(*d*_*i*_^*N*^ + *r*_*i*_^*N*^ *X*_*N*_) = *X*_*N*_ and *d*_*i*_^*N*^ + *r*_*i*_^*N*^ *X*_*N*_≿_*i*_ *X*_{*i*}_ for all *i* ∈ *N*. Let *S* be a coalition and (*d* ^*N*^, *r* ^*N*^) and $\left({\tilde{d}}^{N},{\tilde{r}}^{N}\right)$ be two stochastic payoff vectors of the game Γ. One says (*d* ^*N*^, *r* ^*N*^) dominates $\left({\tilde{d}}^{N},{\tilde{r}}^{N}\right)$ through *S*, $\left(d^{N},r^{N}){≻}_{S}({\tilde{d}}^{N},{\tilde{r}}^{N}\right)$, if $d_{i}^{N}+r_{i}^{N}X_{N}{≻}_{i}{\tilde{d}}_{i}^{N}+{\tilde{r}}_{i}^{N}X_{N}$ for all *i* ∈ *S* and ∑_*i*∈*S*_(*d*_*i*_^*N*^ + *r*_*i*_^*N*^ *X*_*N*_) ≤ *X*_*S*_.

**Definition**

The set of all undominated payoffs for a stochastic cooperative game Γ is called the core of the stochastic cooperative game Γ and denoted by Core(Γ). That is, the payoff *x* = {*x*_1_, *x*_2_,…, *x*_*n*_} of stochastic cooperative game Γ is said to be a core payoff if it satisfies ∑_*i*∈*S*_ *x*_*i*_ ≥ *X*_*S*_ for all *S* ⊂ *N* and ∑_*i*∈*N*_ *x*_*i*_ = *X*_*N*_.

If an allocation is not in the core there is incentive for some agents to leave the coalition. A core solution is desirable because it is stable, but the core of a cooperative game may be empty. In addition, even when the core exists, an allocation in the core may have other undesirable characteristics. In general, it is hard to determine whether the core of a coalitional game exists or not. Even when it does, the more important question is whether the suggested value allocation scheme is actually in the core. While such issues can be important, we avoid them as unpromising in this context. In the sequel we investigate the Owen value of stochastic cooperative games.

## 3. Owen Value of Stochastic Cooperative Games

In this section we consider the Owen value for stochastic cooperative games with coalition structure that can be regarded as an expansion of the Shapley value for the situation when a coalition structure is involved. The Owen value was introduced in Owen [8] via a set of axioms it was determining.

We consider games with coalition structure. A coalition structure *B* = {*B*_1_,…, *B*_*m*_} on a player set *N* is a partition of the player set *N*; that is, *B*_1_ ∪ ⋯*B*_*m*_ = *N* and *B*_*i*_∩*B*_*j*_ = *∅* for *i* ≠ *j*. Denote by *B*_*N*_ a set of all coalition structures on *N*. A coalition value is an operator that assigns a vector of payoffs to any pair (*V*, *B*) of a game *V* and a coalition structure *B* on *N*. More precisely, for any set of game *G*⊆*G*_*N*_ and any set of coalition structures *B*⊆*B*_*N*_, a coalitional value on (*N*, *V*) with a coalition structure from *B* is a mapping *φ* : *G* × *B* → *R* ^*n*^ that associates with each pair (*V*, *B*) of a game *V* ∈ *G* and a coalition structure *B* ∈ *B* a vector *φ*(*V*, *B*) ∈ *R* ^*n*^, where the real number *φ*_*i*_(*V*, *B*) represents the payoff to the player *i* in the game *V* with the coalition structure *B*.

We considers the stochastic cooperative game which induces (*d*, *r*)^*B*^ among coalitions in *B* = {*B*_1_,…, *B*_*m*_}. This game, which is denoted by (*d*, *r*)^*B*^ and called the game between coalitions or intermediate game, is defined formally for every *T*⊆*M* by

(2) $$\begin{array}{c} {\left(d,r\right)}^{B}\left(T\right)=\underset{j\in \left(\cup_{k\in T}B_{k}\right)}{\sum }\left[d_{j}^{\left(\cup_{k\in T}B_{k}\right)}+r_{j}^{\left(\cup_{k\in T}B_{k}\right)}X_{\left(\cup_{k\in T}B_{k}\right)}\right], \end{array}$$

where *M* = {1,2,…, *m*}.

We will use the following axioms to present characterizations of Owen value.

**Definition**

*φ*(*d*, *r*) = {*φ*_1_(*d*, *r*), *φ*_2_(*d*, *r*),…, *φ*_*n*_(*d*, *r*)} ∈ *R* ^*n*^ is called Owen value on (*d*, *r*) if it satisfies the following three axioms.

*Axiom 1* (efficiency). For all (*d*, *r*) ∈ Γ and all *B* ∈ *B*,

(3) $$\begin{array}{c} \underset{i\in N}{\sum }\varphi_{i}\left(\left(d,r\right),B\right)=X_{N}. \end{array}$$

*Axiom 2* (additivity). For all *B* ∈ *B* and all $(d,r),(\tilde{d},\tilde{r})\in \Gamma$, if there exists $(d+\tilde{d},r+\tilde{r})\in \Gamma$ such that $\sum_{j\in S}[{\left(d+\tilde{d}\right)}_{j}^{S}+{\left(r+\tilde{r}\right)}_{j}^{S}X_{S}]=\sum_{j\in S}[d_{j}^{S}+r_{j}^{S}X_{S}]+\sum_{j\in S}[{\tilde{d}}_{j}^{S}+{\tilde{r}}_{j}^{S}X_{S}]$ for all *S*⊆*N*, then

(4) $$\begin{array}{c} \varphi_{i}\left(\left(d+\tilde{d},r+\tilde{r}\right),B\right)=\varphi_{i}\left(\left(d,r\right),B\right)+\varphi_{i}\left(\left(d,r\right),B\right), \end{array}$$

for all *i* ∈ *N*.

*Axiom 3* (null player). For all (*d*, *r*) ∈ Γ and all *B* ∈ *B*, if *i* is a dummy player in the game (*d*, *r*) (i.e., for each *S*, ∑_*j*∈(*S*∪{*i*})_[*d*_*j*_^(*S*∪{*i*})^ + *r*_*j*_^(*S*∪{*i*})^ *X*_(*S*∪{*i*})_] = ∑_*j*∈*S*_[*d*_*j*_^*S*^ + *r*_*j*_^*S*^ *X*_*S*_]), then

(5) $$\begin{array}{c} \varphi_{i}\left(\left(d,r\right),B\right)=0. \end{array}$$

*Axiom 4* (symmetry in the unions). For all (*d*, *r*) ∈ Γ, for any *B*_*k*_ ∈ *B*, and for any *i*, *j* ∈ *B*_*k*_, if for each *S*⊆*N*∖{*i*, *j*} such as ∑_*l*∈(*S*∪{*i*})_[*d*_*l*_^(*S*∪{*i*})^ + *r*_*l*_^(*S*∪{*i*})^ *X*_(*S*∪{*i*})_] = ∑_*l*∈(*S*∪{*j*})_[*d*_*l*_^(*S*∪{*j*})^ + *r*_*l*_^(*S*∪{*j*})^ *X*_(*S*∪{*j*})_], then

(6) $$\begin{array}{c} \varphi_{i}\left(\left(d,r\right),B\right)=\varphi_{j}\left(\left(d,r\right),B\right). \end{array}$$

*Axiom 5* (symmetry across the unions). For all (*d*, *r*) ∈ Γ and for any *B*_*u*_, *B*_*s*_ ∈ *B*, if for all *T*⊆*M*∖{*u*, *s*} which satisfies (*d*, *r*)^*B*^(*T* ∪ {*u*}) = (*d*, *r*)^*B*^(*T* ∪ {*s*}), then

(7) $$\begin{array}{c} \underset{i\in B_{u}}{\sum }\varphi_{i}\left(\left(d,r\right),B\right)=\underset{i\in B_{s}}{\sum }\varphi_{i}\left(\left(d,r\right),B\right). \end{array}$$

**Theorem 5**

Let *N* = {1,2,…, *n*} be a set of *n* players; then the unique Owen value of the stochastic cooperative games Γ is

(8)
Ow
i((d,r),B) =∑Q⊆(M∖{p})∑ S⊆Bp∖{i}q!(m−q−1)!s!(bp−s−1)!m!bp!  ×{∑j∈(∪t∈QBt∪S∪{i})[dj(∪t∈QBt∪S∪{i})+rj(∪t∈QBt∪S∪{i})X(∪t∈QBt∪S∪{i})]    −∑j∈(∪t∈QBt∪S)[dj(∪t∈QBt∪S)+rj(∪t∈QBt∪S)X(∪t∈QBt∪S)]},

for all *i* ∈ *N*, where *p* is such that *i* ∈ *B*_*p*_ ∈ *B* and *q* = |*Q*|, *b*_*p*_ = |*B*_*p*_|, *s* = |*S*|.

**Proof**

In this proof, we will prove two key issues: (1) the existence of the Owen value and (2) the uniqueness of the Owen value.

(*1) Proof of Existence*

*Axiom 1* (efficiency). Consider

(9) ∑i∈NOwi((d,r),B) =∑i∈N∑ Q⊆(M∖{p})∑ S⊆Bp∖{i}q!(m−q−1)!s!(bp−s−1)!m!bp!  ×{∑j∈(∪t∈QBt∪S∪{i})[dj(∪t∈QBt∪S∪{i})+rj(∪t∈QBt∪S∪{i})X(∪t∈QBt∪S∪{i})]    −∑j∈(∪t∈QBt∪S)[dj(∪t∈QBt∪S)+rj(∪t∈QBt∪S)X(∪t∈QBt∪S)]} =1m!∑π′∑ i∈N∑ Q⊆(M∖{p})∑ S⊆Bp∖{i}q!(m−q−1)!s!(bp−s−1)!m!bp!  ×{∑j∈(∪t∈QBt∪S∪{i})[dj(∪t∈QBt∪S∪{i})+rj(∪t∈QBt∪S∪{i})X(∪t∈QBt∪S∪{i})]    −∑j∈(∪t∈QBt∪S)[dj(∪t∈QBt∪S)+rj(∪t∈QBt∪S)X(∪t∈QBt∪S)]} =1m!∑π′∑ k=1m∑ i∈Bπk′1bπk′!∑ππk′  ×{∑j∈(⋃t=1k−1Bπt′∪Sππk′i∪{i})[dj(⋃t=1k−1Bπt′∪Sππk′i∪{i})+rj(⋃t=1k−1Bπt′∪Sππk′i∪{i})×X(⋃t=1k−1Bπt′∪Sππk′i∪{i})]    −∑j∈(⋃t=1k−1Bπt′∪Sππk′i)[dj(⋃t=1k−1Bπt′∪Sππk′i)+rj(⋃t=1k−1Bπt′∪Sππk′i)X(⋃t=1k−1Bπt′∪Sππk′i)]},∑i∈NOwi((d,r),B) =1m!∑π′∑ k=1m1bπk′!∑ππk′∑ i∈Bπk′  ×{∑j∈(⋃t=1k−1Bπt′∪Sππk′i∪{i})[dj(⋃t=1k−1Bπt′∪Sππk′i∪{i})+rj(⋃t=1k−1Bπt′∪Sππk′i∪{i})×X(⋃t=1k−1Bπt′∪Sππk′i∪{i})]    −∑j∈(⋃t=1k−1Bπt′∪Sππk′i)[dj(⋃t=1k−1Bπt′∪Sππk′i)+rj(⋃t=1k−1Bπt′∪Sππk′i)×X(⋃t=1k−1Bπt′∪Sππk′i)]} =1m!∑π′∑k=1m1bπk′!∑ππk′[(d,r)B(⋃t=1kBπt′)−(d,r)B(⋃t=1k−1Bπt′)] =1m!∑π′∑k=1m[(d,r)B(⋃t=1kBπt′)−(d,r)B(⋃t=1k−1Bπt′)] =1m!∑π′(d,r)B(⋃t=1mBπt′)=1m!∑π′XN=XN.

*Axiom 2* (additivity). It is easy to see from (8) that Ow_*i*_((*d*, *r*), *B*) is a linear function for (*d*, *r*); it obviously satisfies additivity.

*Axiom 3* (null player). For (*d*, *r*) ∈ Γ and *B* ∈ *B*, we suppose that *i* is a dummy player in the game (*d*, *r*); then

(10) ∑j∈(∪t∈QBt∪S∪{i})[dj(∪t∈QBt∪S∪{i})+rj(∪t∈QBt∪S∪{i})X(∪t∈QBt∪S∪{i})] =∑j∈(∪t∈QBt∪S)[dj(∪t∈QBt∪S)+rj(∪t∈QBt∪S)X(∪t∈QBt∪S)],

for any *i* ∈ *B*_*p*_ ∈ *B*, *S*⊆*B*_*p*_, and *Q*⊆(*M*∖{*p*}); that is,

(11) ∑j∈(∪t∈QBt∪S∪{i})[dj(∪t∈QBt∪S∪{i})+rj(∪t∈QBt∪S∪{i})X(∪t∈QBt∪S∪{i})] −∑j∈(∪t∈QBt∪S)[dj(∪t∈QBt∪S)+rj(∪t∈QBt∪S)X(∪t∈QBt∪S)]=0.

It is easy to see from (8) that

(12) $$\begin{array}{c} {\text{Ow}}_{i}\left(\left(d,r\right),B\right)=0. \end{array}$$

*Axiom 4* (symmetry in the unions). For all (*d*, *r*) ∈ Γ, for any *B*_*k*_ ∈ *B*, and for any *i*, *j* ∈ *B*_*k*_, if for each *S*⊆*N*∖{*i*, *j*} which satisfies ∑_*l*∈(*S*∪{*i*})_[*d*_*l*_^(*S*∪{*i*})^ + *r*_*l*_^(*S*∪{*i*})^ *X*_(*S*∪{*i*})_] = ∑_*l*∈(*S*∪{*j*})_[*d*_*l*_^(*S*∪{*j*})^ + *r*_*l*_^(*S*∪{*j*})^ *X*_(*S*∪{*j*})_], then

(13) ∑l∈(∪t∈QBt∪S∪{i})[dl(∪t∈QBt∪S∪{i})+rl(∪t∈QBt∪S∪{i})X(∪t∈QBt∪S∪{i})] =∑l∈(∪t∈QBt∪S∪{j})[dl(∪t∈QBt∪S∪{j})+rl(∪t∈QBt∪S∪{j})X(∪t∈QBt∪S∪{j})],

for any *i*, *j* ∈ *B*_*p*_ ∈ *B*, *S*⊆*B*_*p*_, and *Q*⊆(*M*∖{*p*}).

From (8) we have that

(14) $$\begin{array}{c} {\text{Ow}}_{i}\left(\left(d,r\right),B\right)={\text{Ow}}_{j}\left(\left(d,r\right),B\right). \end{array}$$

*Axiom 5* (symmetry across the unions). From the assumption of Axiom 5, we have that

(15) $$\begin{array}{c} {\left(d,r\right)}^{B}\left(T\cup \left\{u\right\}\right)={\left(d,r\right)}^{B}\left(T\cup \left\{s\right\}\right), \end{array}$$

for all *T*⊆*M*∖{*u*, *s*}; In particular,

(16) $$\begin{array}{c} {\left(d,r\right)}^{B}\left(M\setminus \left\{u\right\}\right)={\left(d,r\right)}^{B}\left(M\setminus \left\{s\right\}\right), \end{array}$$

when *T* = *M*∖{*u*, *s*}, then we obtain

(17) ∑i∈BuOwi((d,r),B) =∑i∈Bu∑ Q⊆(M∖{u})∑ S⊆Bu∖{i}q!(m−q−1)!s!(bu−s−1)!m!bu!  ×{∑j∈(∪t∈QBt∪S∪{i})[dj(∪t∈QBt∪S∪{i})+rj(∪t∈QBt∪S∪{i})×X(∪t∈QBt∪S∪{i})]    −∑j∈(∪t∈QBt∪S)[dj(∪t∈QBt∪S)+rj(∪t∈QBt∪S)X(∪t∈QBt∪S)]} =∑Q⊆(M∖{u})q!(m−q−1)!m!bu!∑πu∑ i∈Bu  ×{∑j∈(∪t∈QBt∪Sπui∪{i})[dj(∪t∈QBt∪Sπui∪{i})+rj(∪t∈QBt∪Sπui∪{i})X(∪t∈QBt∪Sπui∪{i})]    −∑j∈(∪t∈QBt∪Sπui)[dj(∪t∈QBt∪Sπui)+rj(∪t∈QBt∪Sπui)X(∪t∈QBt∪Sπui)]} =∑Q⊆[M∖(u∪s)]q!(m−q−1)!m!bu!∑πu∑ i∈Bu  ×{∑j∈(∪t∈QBt∪Sπui∪{i})[dj(∪t∈QBt∪Sπui∪{i})+rj(∪t∈QBt∪Sπui∪{i})X(∪t∈QBt∪Sπui∪{i})]    −∑j∈(∪t∈QBt∪Sπui)[dj(∪t∈QBt∪Sπui)+rj(∪t∈QBt∪Sπui)X(∪t∈QBt∪Sπui)]}  +∑[M∖(u∪s)]⊂Q⊆[M∖(u)]q!(m−q−1)!m!bu!∑πu∑ i∈Bu  ×{∑j∈(∪t∈QBt∪Sπui∪{i})[dj(∪t∈QBt∪Sπui∪{i})+rj(∪t∈QBt∪Sπui∪{i})X(∪t∈QBt∪Sπui∪{i})]    −∑j∈(∪t∈QBt∪Sπui)[dj(∪t∈QBt∪Sπui)+rj(∪t∈QBt∪Sπui)X(∪t∈QBt∪Sπui)]} =∑Q⊆(M∖{u,s})q!(m−q−1)!m![(d,r)B(Q∪{u})−(d,r)B(Q)]  +∑Q=(M∖u)1m[(d,r)B(M)−(d,r)B(M∖u)],

where *π*_*k*_ is any permutation in *B*_*k*_ and *S*_*π*_*k*__^*i*^ = {*j* ∈ *B*_*k*_ | *π*_*k*_(*j*) < *π*_*k*_(*i*)} is the set of players preceding player *i* in the permutation *π*_*k*_, for all *k* ∈ *M*.

From (15), (16), and (17), we have that

(18) ∑i∈BuOwi((d,r),B) =∑Q⊆(M∖{u,s})q!(m−q−1)!m!  ×[(d,r)B(Q∪{s})−(d,r)B(Q)]  +∑Q=(M∖s)1m[(d,r)B(M)−(d,r)B(M∖s)] =∑Q⊆(M∖{u,s})q!(m−q−1)!m!bs!  ×∑πs{∑j∈(∪t∈QBt∪Bs)[dj(∪t∈QBt∪Bs)+rj(∪t∈QBt∪Bs)X(∪t∈QBt∪Bs)]     −∑j∈(∪t∈QBt)[dj(∪t∈QBt)+rj(∪t∈QBt)X(∪t∈QBt)]}  +∑Q=(M∖s)1mbs!  ×∑πs{∑j∈N[djN+rjNXN]     −∑j∈(N∖Bs)[dj(N∖Bs)+rj(N∖Bs)X(N∖Bs)]} =∑i∈Bs∑ Q⊆(M∖{s})∑ S⊆Bs∖{i}q!(m−q−1)!s!(bs−s−1)!m!bs!  ×{∑j∈(∪t∈QBt∪S∪{i})[dj(∪t∈QBt∪S∪{i})+rj(∪t∈QBt∪S∪{i})X(∪t∈QBt∪S∪{i})]    −∑j∈(∪t∈QBt∪S)[dj(∪t∈QBt∪S)+rj(∪t∈QBt∪S)X(∪t∈QBt∪S)]} =∑i∈BsOwi((d,r),B).

(*2) Proof of Uniqueness*. Let *φ* be a coalitional value which have efficiency, additivity, null player, and symmetry in the unions and across the unions, and let *B* ∈ *B*_*N*_; then *φ* is defined on Γ × *B*_*N*_. Any stochastic cooperative game (*d*, *r*) ∈ Γ can be presented via unanimity basis {*ω*_*T*_}_*∅*≠*T*⊆*N*_:

(19) $$\begin{array}{c} \left(d,r\right)=\underset{\emptyset \neq T\subseteq N}{\sum }c_{T}\omega_{T}, \end{array}$$

where *c*_*T*_ = ∑_*U*⊆*T*_(−1)^|*T*|−|*U*|^(*d* ^*U*^, *r* ^*U*^), and

(20) $$\begin{array}{c} \omega_{T}\left(S\right)=\{\begin{array}{cc} 1, & S⊇T,\\ 0, & \text{otherwise}. \end{array} \end{array}$$

The Owen value in the unanimity game {*ω*_*T*_} with a coalition structure *B* is equal to

(21) $$\begin{array}{c} {\text{Ow}}_{i}\left(\omega_{T},B\right)=\{\begin{array}{cc} \frac{1}{|B\left(i\right)\cap T|m_{T}}, & i\in T,\\ 0, & i\in N\setminus T, \end{array} \end{array}$$

where *B*(*i*) is the element of the coalition structure *B* that contains player *i* and *m*_*T*_ is equal to the number of coalitions in *B* that have a nonempty intersection with *T*; that is, *B*(*i*) = *B*_*k*_ ∈ *B* : *B*_*k*_∋*i* and *m*_*T*_ = |{*k* ∈ *M* : *B*_*k*_∩*T* ≠ *∅*}|. Because of its additivity property the Owen value in any stochastic cooperative game (*d*, *r*) with a coalition structure *B* can be equivalently expressed as

(22) $$\begin{array}{c} {\text{Ow}}_{i}\left(\omega_{T},B\right)=\underset{\emptyset \neq T\subseteq N:T∋i}{\sum }\frac{c_{T}}{|B\left(i\right)\cap T|m_{T}}. \end{array}$$

Let the index *I* of a stochastic cooperative game (*d*, *r*) ∈ Γ be the minimum number of terms under summation in (20); then

(23) $$\begin{array}{c} \left(d,r\right)=\overset{I}{\underset{r=1}{\sum }}c_{T_{r}}\omega_{T_{r}}, \end{array}$$

where all *c*_*T*_*r*__ ≠ 0. We proceed with the remaining part of the proof by induction on this index *I*.

If *I* = 0, then (*d*, *r*) is identically zero on all coalitions. All players in both games (*d*, *r*) and (*d*, *r*)^*B*^ are symmetric. Therefore, by symmetry across coalitions for all *k*, *l* ∈ *M*,

(24) $$\begin{array}{c} \underset{i\in B_{k}}{\sum }\psi_{i}\left(\left(d,r\right),B\right)=\underset{i\in B_{l}}{\sum }\psi_{i}\left(\left(d,r\right),B\right). \end{array}$$

But

(25) $$\begin{array}{c} \underset{k\in M}{\sum }\underset{\;i\in B_{k}}{\sum }\psi_{i}\left(\left(d,r\right),B\right)=\underset{i\in N}{\sum }\psi_{i}\left(\left(d,r\right),B\right), \end{array}$$

and by efficiency

(26) $$\begin{array}{c} \underset{i\in N}{\sum }\psi_{i}\left(\left(d,r\right),B\right)=0. \end{array}$$

Thus, for all *k* ∈ *M*,

(27) $$\begin{array}{c} \underset{i\in B_{k}}{\sum }\psi_{i}\left(\left(d,r\right),B\right)=0. \end{array}$$

By symmetry within coalitions it follows that, for all *i* ∈ *N*,

(28) $$\begin{array}{c} \psi_{i}\left(\left(d,r\right),B\right)=0; \end{array}$$

that is

(29) $$\begin{array}{c} \psi_{i}\left(\left(d,r\right),B\right)={\text{Ow}}_{i}\left(\left(d,r\right),B\right)=0. \end{array}$$

Assume now that *ψ*((*d*, *r*), *B*) is the Owen value whenever the index of (*d*, *r*) ∈ Γ is at most *I*, and consider some (*d*, *r*) ∈ Γ with the index being equal to *I* + 1. Let *T* = ∩_*r*=1_^*I*+1^ *T*_*r*_ and *i* ∈ *N*∖*T*. Consider the game

(30) $$\begin{array}{c} {\left(d,r\right)}^{i}=\underset{r:T_{r}∋i}{\sum }c_{T_{r}}\omega_{T_{r}}. \end{array}$$

Obviously, the index of (*d*, *r*)^*i*^ is at most *I* and

(31) $$\begin{array}{c} \left(d,r\right)={\left(d,r\right)}^{i}+c_{T}\omega_{T}; \end{array}$$

therefore, by induction hypothesis,

(32) $$\begin{array}{c} \psi_{i}\left({\left(d,r\right)}^{i},B\right)={\text{Ow}}_{i}\left({\left(d,r\right)}^{i},B\right), \end{array}$$

for all *i* ∈ *N*∖*T*.

Using the additivity of the *ψ* and the Owen value, we have

(33) $$\begin{array}{c} \psi_{i}\left(\left(d,r\right),B\right)=\psi_{i}\left({\left(d,r\right)}^{i},B\right)+\psi_{i}\left(c_{T}\omega_{T},B\right),\\ {\text{Ow}}_{i}\left(\left(d,r\right),B\right)={\text{Ow}}_{i}\left({\left(d,r\right)}^{i},B\right)+{\text{Ow}}_{i}\left(c_{T}\omega_{T},B\right), \end{array}$$

for all *i* ∈ *N*∖*T*.

By the definition of the unanimity game, we have that

(34) $$\begin{array}{c} \psi_{i}\left(c_{T}\omega_{T},B\right)={\text{Ow}}_{i}\left(c_{T}\omega_{T},B\right)=0, \end{array}$$

for all *i* ∈ *N*∖*T*.

From (32), (33) and (34), we have that

(35) $$\begin{array}{c} \psi_{i}\left(\left(d,r\right),B\right)={\text{Ow}}_{i}\left(\left(d,r\right),B\right), \end{array}$$

for all *i* ∈ *N*∖*T*.

If *T* ≠ *∅* then to complete the proof it is enough to show that the last equality is true for all *i* ∈ *T* as well. Consider *T* with relevance to a coalition structure *B* and denote that

(36) $$\begin{array}{c} M_{T}=\left\{k\in M\;|\;B_{k}\cap T\neq \emptyset ,B_{k}\in B\right\}. \end{array}$$

Notice that if *T* ≠ *∅* then *M*_*T*_ ≠ *∅* and all players *k*, *l* ∈ *M*_*T*_ are symmetric in the stochastic cooperative game (*d*, *r*)^*B*^. By symmetry among coalitions for both values *ψ* and Owen value, for all players *k*, *l* ∈ *M*_*T*_,

(37) $$\begin{array}{c} \underset{i\in B_{k}}{\sum }\psi_{i}\left(\left(d,r\right),B\right)=\underset{i\in B_{l}}{\sum }\psi_{i}\left(\left(d,r\right),B\right),\\ \underset{i\in B_{k}}{\sum }{\text{Ow}}_{i}\left(\left(d,r\right),B\right)=\underset{i\in B_{l}}{\sum }{\text{Ow}}_{i}\left(\left(d,r\right),B\right). \end{array}$$

Therefore, because of efficiency of both values and equality (37) it follows that

(38) $$\begin{array}{c} \underset{i\in B_{k}}{\sum }\psi_{i}\left(\left(d,r\right),B\right)=\underset{i\in B_{k}}{\sum }{\text{Ow}}_{i}\left(\left(d,r\right),B\right), \end{array}$$

for all *k* ∈ *M*_*T*_.

Using the equality (35), we have

(39) $$\begin{array}{c} \underset{i\in \left[B_{k}\cap \left(N\setminus T\right)\right]}{\sum }\psi_{i}\left(\left(d,r\right),B\right)=\underset{i\in \left[B_{k}\cap \left(N\setminus T\right)\right]}{\sum }{\text{Ow}}_{i}\left(\left(d,r\right),B\right), \end{array}$$

for all *k* ∈ ({1,2,…, *m*}∖*M*_*T*_); then

(40) $$\begin{array}{c} \underset{i\in B_{k}\cap T}{\sum }\psi_{i}\left(\left(d,r\right),B\right)=\underset{i\in B_{k}\cap T}{\sum }{\text{Ow}}_{i}\left(\left(d,r\right),B\right), \end{array}$$

for all *k* ∈ *M*_*T*_.

But all players *i* ∈ *T* are symmetric in the game (*d*, *r*). Hence, by symmetry within coalitions, for all *i*, *j* ∈ *B*_*k*_∩*T*, *K* ∈ *M*_*T*_,

(41) $$\begin{array}{c} \psi_{i}\left(\left(d,r\right),B\right)=\psi_{j}\left(\left(d,r\right),B\right),\\ {\text{Ow}}_{i}\left(\left(d,r\right),B\right)={\text{Ow}}_{j}\left(\left(d,r\right),B\right). \end{array}$$

Thus

(42) $$\begin{array}{c} \psi_{i}\left(\left(d,r\right),B\right)={\text{Ow}}_{i}\left(\left(d,r\right),B\right), \end{array}$$

for all *i* ∈ *T*.

From (35) and (42), we have that

(43) $$\begin{array}{c} \psi_{i}\left(\left(d,r\right),B\right)={\text{Ow}}_{i}\left(\left(d,r\right),B\right), \end{array}$$

for all *i* ∈ *N*. This completes the proof.

## 4. Conclusion

In this paper, we consider stochastic cooperative game and give it the definition of the Owen value, which is obtained by extending the classical case. Then we provide explicit expression for the Owen value of the stochastic cooperative game and discuss its existence and uniqueness. In future, we will explore the applications of Owen value of stochastic cooperative game in economy.
